# Supplementary material for: Nitrogen nutrition contributes to plant fertility by affecting meiosis initiation
Source: Nat Commun. 2022 Jan 25;13:485. doi: 10.1038/s41467-022-28173-3 (PMC8789853; doi:10.1038/s41467-022-28173-3)
Supplement: Supplementary file 1 — Supplementary Information [file 41467_2022_28173_MOESM1_ESM.pdf]

Supplementary Infromation

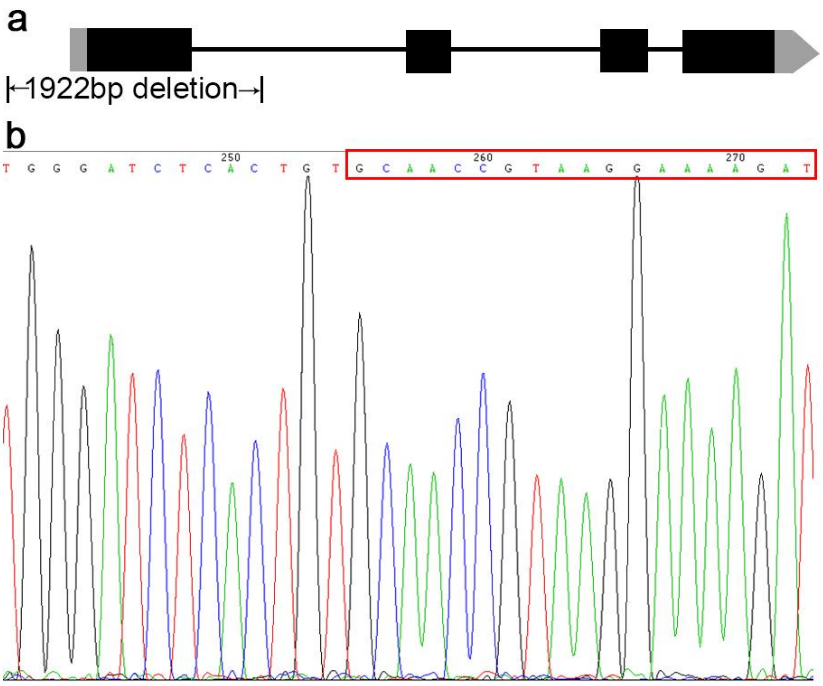

Supplementary Figure 1. **Mutation site of the *etfβ* allele.**

(a) The structure of the *ETFβ* gene. The black boxes indicate exons. The deletion of 1922 bp included the 5'-untranslated region (UTR) and the first exon. (b) The detection of the large fragment deletion in *ETFβ*. We use *ETFβ*-a-ID-F as the sequencing primer to detect the deleted DNA fragment in the mutant. The *ETFβ*-a-ID-F was highlighted within a red box.

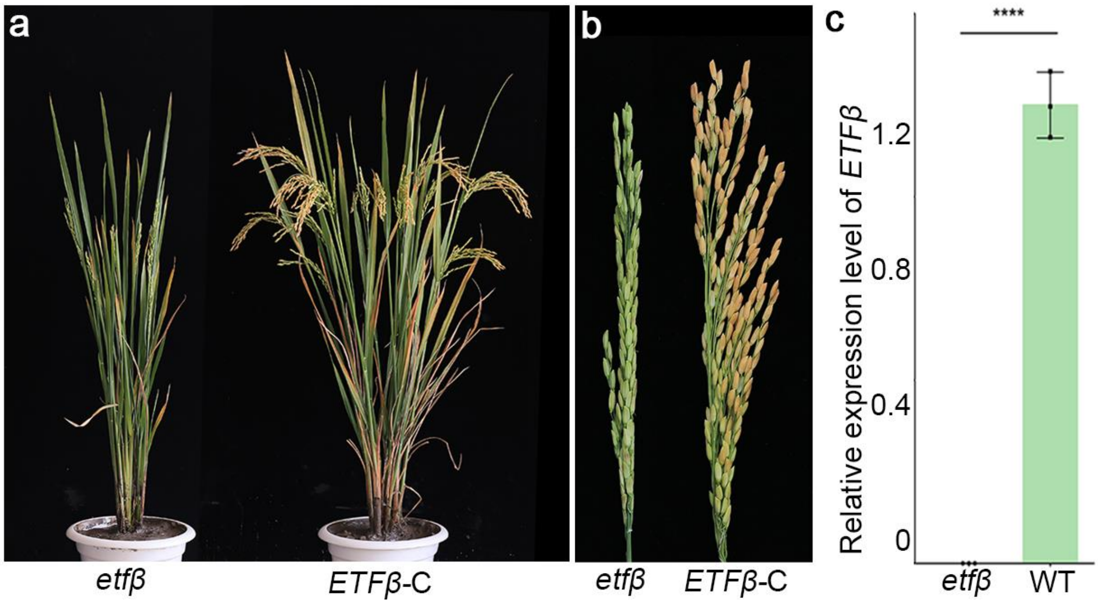

Supplementary Figure 2. **Characterization of the rice sterile mutant *etfβ* and its complenmentary plant *ETFβ*-C.**

(a) Morphology of the rice sterile mutant *etfβ* and *ETFβ*-C after heading. (b) Panicle morphology of the *etfβ* and *ETFβ*-C. We transgenically expressed the *ETFβ* gene in *etfβ* via the transformation of A plasmid containing the entire open reading frame (ORF), 5.0-kb upstream region, and 1.8-kb downstream region of *ETFβ* into the *etfβ* mutant rescued the fertility. (c) Relative expression of *ETFβ* in *etfβ* and WT. Values are means ± standard deviation (SD) of three independent biological replicates in real-time PCR. Significance was determined by two-tailed Student's *t*-test. **, $p < 0.01$; ***, $p < 0.001$; ****, $p < 0.0001$.

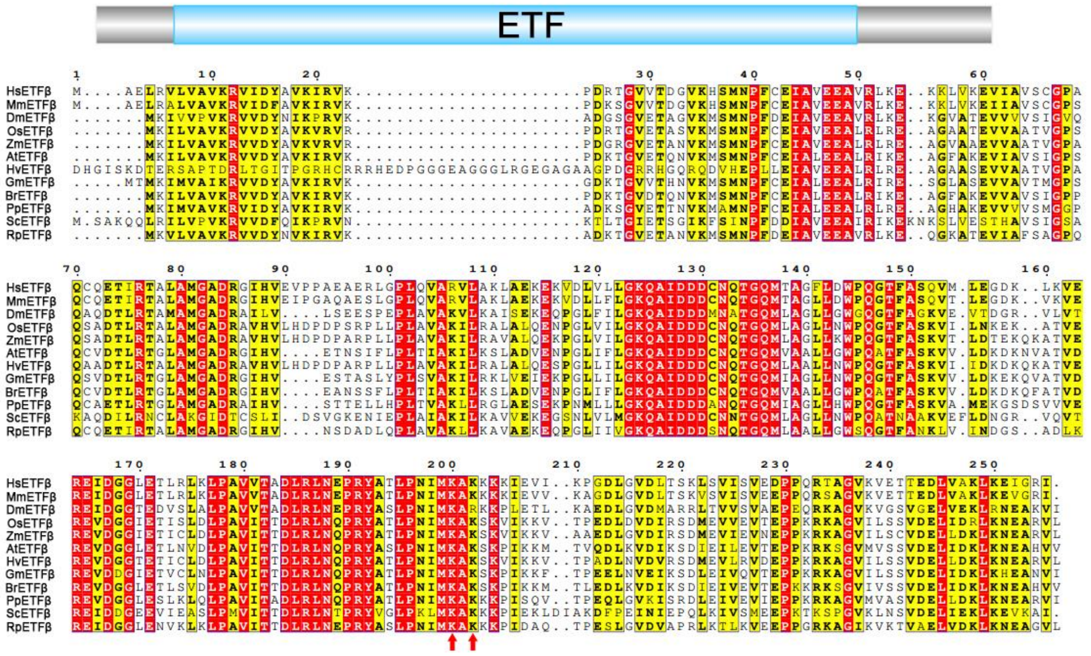

Supplementary Figure 3. **Full length alignment of Electron Transfer Flavorprotein subunit β (ETFβ) proteins.**

ETFβ is a nucleus-encoded mitochondria protein, containing a single electron transfer (ETF) domain (amino acids 23–215). Alignments of the amino acid sequences of Rice ETFβ to its homologues from bacteria to human show high overall similarities. Two adjacent highly conservative lysine residues(marked with red arrows), Lys200 and Lys203 in human ETFβ both *in vitro* and in cells were found to be specifically methylated by ETFβ-KMT. At, *Arabidopsis thaliana*; Br, *Brassica rapa*; Dm, *Drosophila melanogaster*; Gm, *Glycine max*; Hs, *Homo sapiens*; Hv, *Hordeum vulgare*; Mm, *Mus musculus*; Os, *Oryza sativa*; Pp, *Physcomitrella patens*; Rp, *Rhodospirillaceae bacterium*; Sc, *Saccharomyces cerevisiae*; Zm, *Zea mays*.

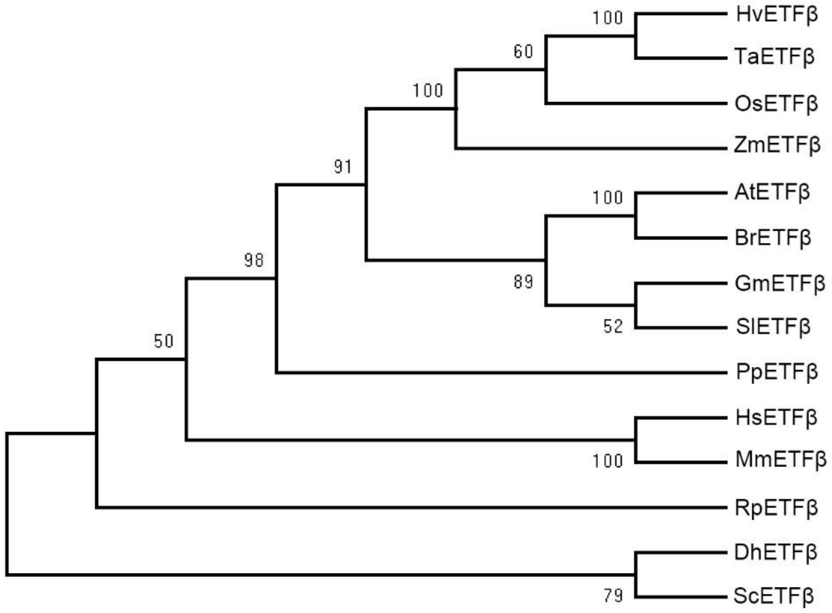

Supplementary Figure 4. **Phylogenetic tree of the ETFβ proteins and its orthologs in different organisms.**

The phylogenetic tree was constructed using MEGA v.5.2. At, *Arabidopsis thaliana*; Br, *Brassica rapa*; Dh, *Drosophila hydei*; Gm, *Glycine max*; Hs, *Homo sapiens*; Hv, *Hordeum vulgare*; Mm, *Mus musculus*; Os, *Oryza sativa*; Pp, *Physcomitrella patens*; Rp, *Rhodospirillaceae bacterium*; Sc, *Saccharomyces cerevisiae*; Sl, *Solanum lycopersicum*; Zm, *Zea mays*.

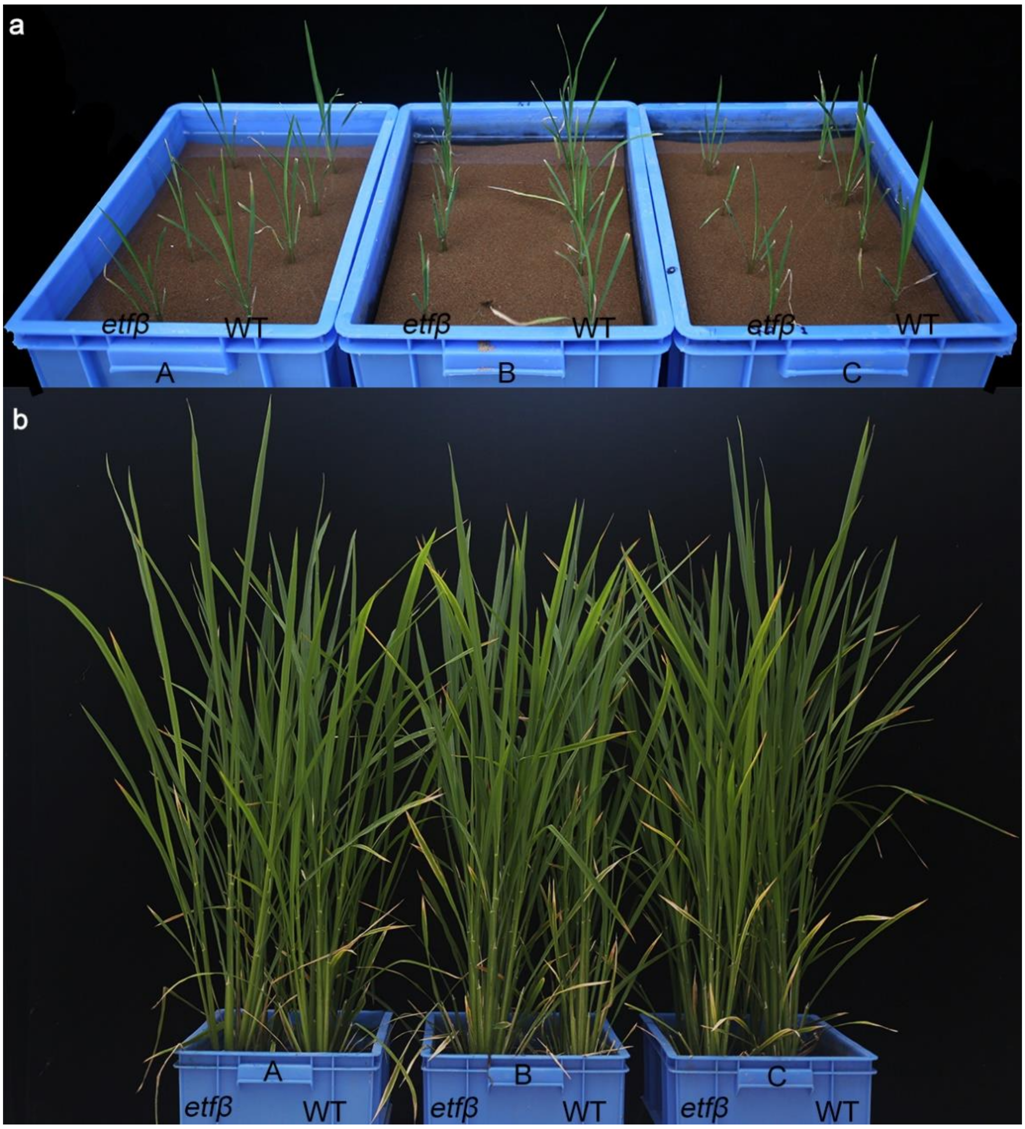

Supplementary Figure 5. **Plants were planted in porous ceramic cultivated with nutriental solution from seeding stage.**

(a) Whole plants at seeding stage. (b), Whole plants after 50 days for vegetative growth treated with three nutritional conditions (A, B and C). *etfβ* plants are in the left panel of each pot, and WT plants are in the right panel of each pot.

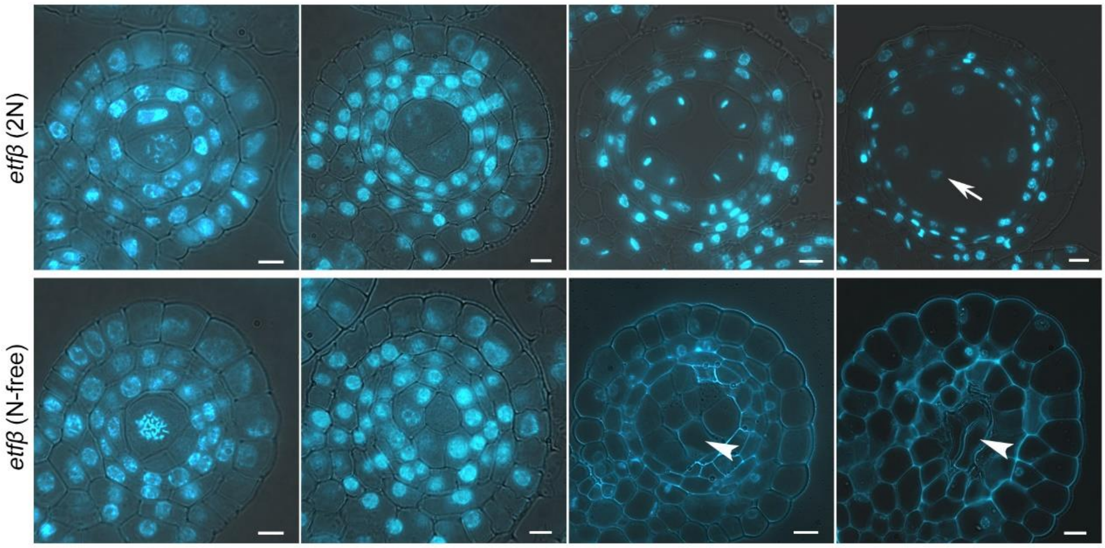

Supplementary Figure 6. Transverse sections of *etfβ* (2N) and *etfβ* (free) anthers at various development stages.

Matured pollen grains formed normally in *etfβ* (2N) anther locules, while a narrow cavity in the *etfβ* (N-free) anther locule. The arrow points to Matured pollen grains, and arrowheads point to abnormal apoptosis in the center of locules. Slides were stained with DAPI. Scale bars, 5 μm.

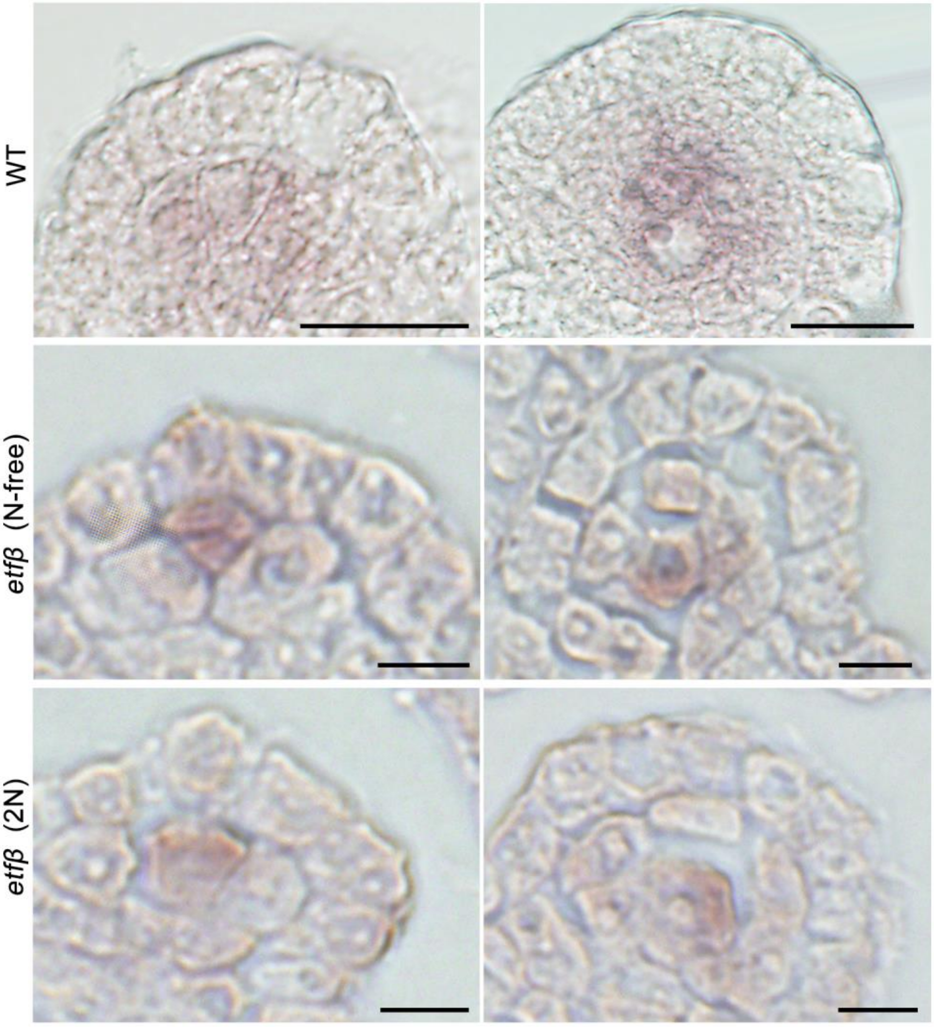

Supplementary Figure 7. ***In situ*** **expression analysis of** ***MEL1*** **in WT,** ***etfβ*** **(N-free) and** ***etfβ*** **(2N) anthers at one-layer stage and two-layer stage.**

Scale bars, 5 μm.

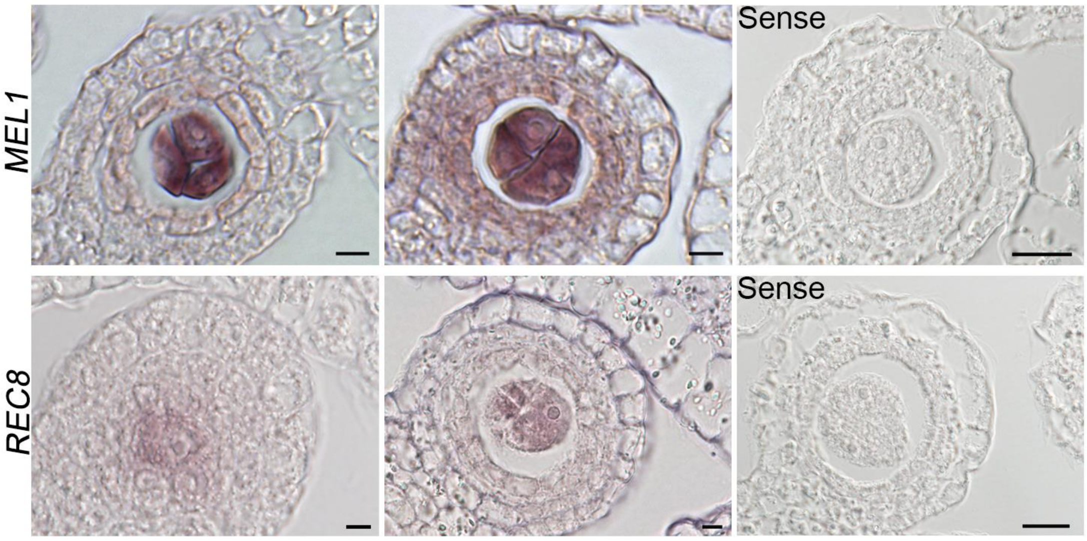

Supplementary Figure 8. Expression patterns of *MEL1* and *REC8* in WT anthers.

RNA *in situ* hybridization was performed to detect the expression patterns of *MEL1* and *REC8* in WT. Hybridization with sense *MEL1* and *REC8* transcript provided the negative control. In WT anthers under N-free condition, a faint *MEL1* mRNA signal was first detected in PSCs. Afterwards the signal became very strong in SCs and was persistently strong in the early PMCs. REC8 is a member of meiosis-specific cohesion, and *REC8* expresses preferentially in SCs and microsporocytes of wild-type. Scale bars, 5 um.

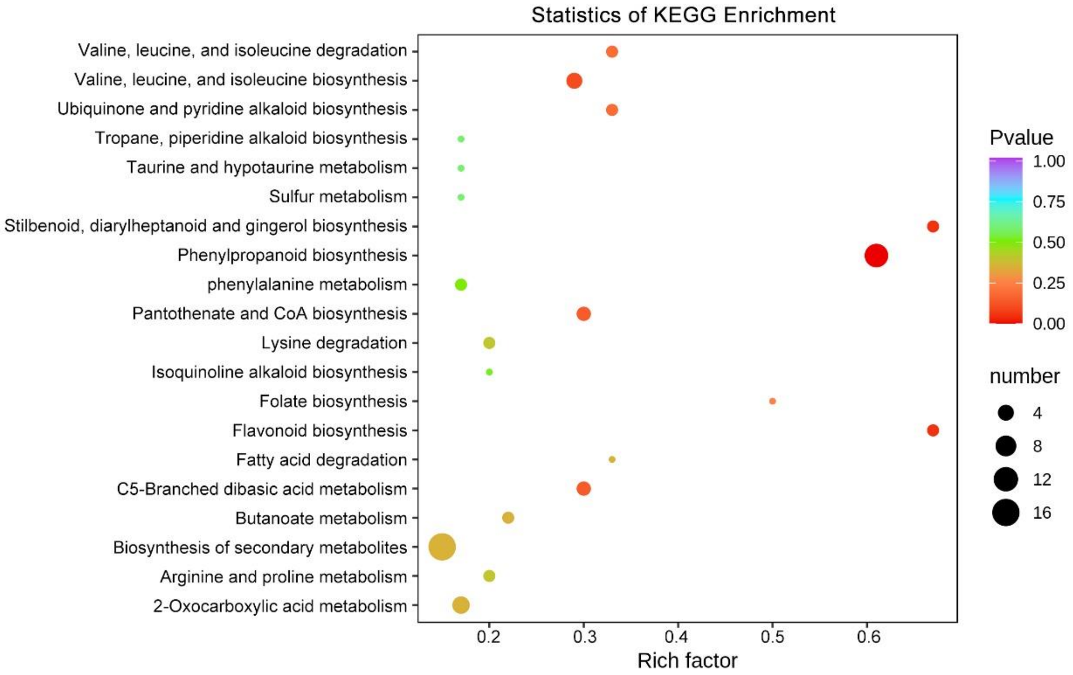

Supplementary Figure 9. The KEGG pathways of differential metabolites related to the metabolism and synthesis of amino acids, especially for branched amino acids of 9 cm panicles from WT and *etfβ* under the N-free condition.

Pathways with significantly regulated metabolites mapped to were then fed into MSEA (metabolite sets enrichment analysis), their significance was determined by hypergeometric test's p-values. The abscissa indicates the rich factor corresponding to each path, the ordinate does not indicate the path name, and the color of the point indicates the pvalue. The redder the point, the richer it is. The size of the dot represents the number of metabolites enriched in significant difference.

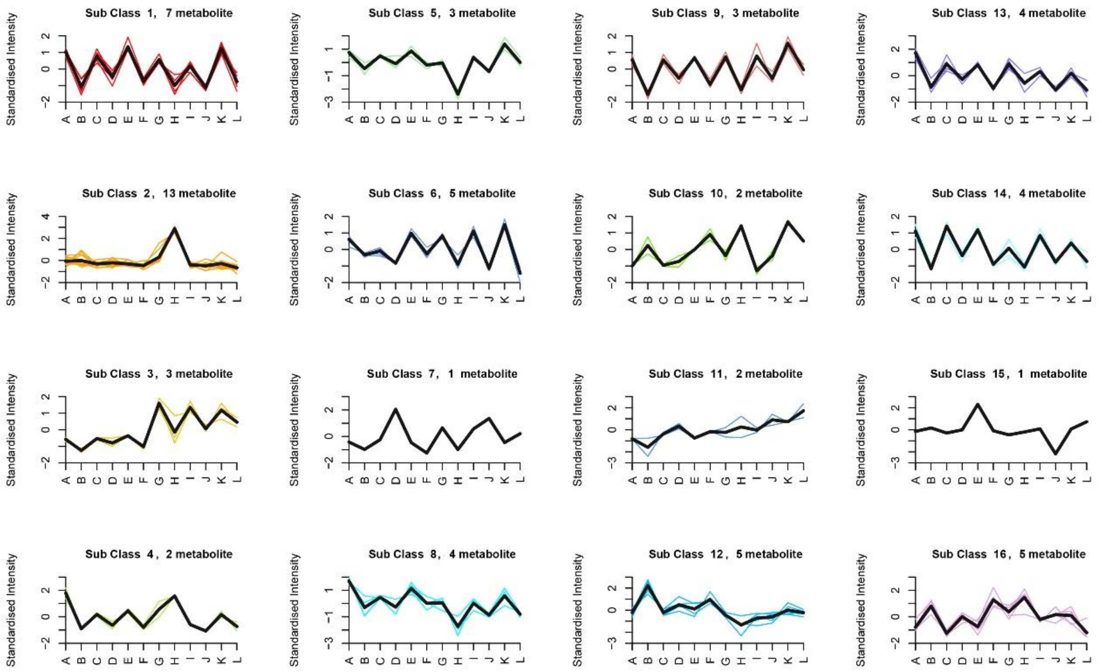

Supplementary Figure 10. Kmeans cluster information tables of differential metabolites. Differential metabolites were calssified into 16 subclasses in the kmeans cluster according to the trends of their contents in 12 groups of samples. A-F, WT; G-L, *etfβ*. A, 0-9; B, 0-18; C, 1-9; D, 1-18; E, 2-9; F, 2-18; G, 0-9; H, 0-18; I, 1-9; J, 1-18; K, 2-9; L, 2-18. The first digit of each group name represents the nitrogen gradient, the second digit represents the length of panicles, and the last letter represents the genotype AA and aa, respectively. A sharp peak can be seen at G in subclsss 2 and subclsss 3, which include leucine, isoleucine, glutraic acid, 2-hydroxyglutaric acid, and their derivatives, corresponding to the metabolic defects of leucine and isoleucine and 2-hydroxyglutaric acid.

Supplementary Table 1. Primers for the markers used in map-based cloning.

Marker	Forward primer sequence	Reverse primer sequence	BAC
M1	ATGATACCTAGATGTGCGAT	CCGAAATAACGTCATTCTGC	AL606654
M2	GGAGGAGAGTTTTTGGGTTC	GCCTTAGTGGAGAAATCCGA	AL606997
M3	CAGAGAGAAGTCTTAAGACATG	TACATTACGTTTCCGATCGA	CR933498
M4	CTGTGTGTCCAGATTCATAGC	CGAAATCTTGTTTCGGCACT	CR933498
M5	CTCCATTATGAGTCTATGAC	GTTGAATGGCTCGGAGGGAA	AL606993
M6	CCGTTTGTACATGTGTTATCTG	GCCACAAATCAAACCACCAAATG	AL662934
M7	GGGGGTAATTCATACTTTTTCC	GCATATTGGCCTGCATAATGTT	AL731620
M8	AACACACGAAGTGACTTTTAGG	GAAGGAGATAGAGACCTGAC	AL662994

Supplementary Table 2. Primers for plasmid construction and RT-PCR.

Primer name	Primer sequence	Description
ETFβ-C-F	CTTGTTGCTCGCTCACCATC	Genetic complementation
ETFβ-C-R	GATCGAACGCAAGCTAGAC	
ETFβ-GFP-F	AGCCCAAGCTTGTCGACG<br>ATGAAGATCCTAGTGGCGGT	Subcellular localization
ETFβ-GFP-R	CTCGCCCTTGCTCACCATCTTAGCTGCTG<br>CAGCTTCTAAAACACGGGCTTCATTTTTC	
ETFβ-A-ID-F	CTCGCAACTCTCCTCTCTTC	Genotype identification
ETFβ-A-ID-R	ACTAGGATCTTCATGCCTCG	
ETFβ-a-ID-F	GGAGCATTAGGTCTAAAGG	Genotype identification
ETFβ-a-ID-R	ATCTTTTCCTTACGGTTGC	
ETFβ-RT-F	GCTGGGTTGCTTAATTGGC	Real-Time PCR
ETFβ-RT-R	CTCAAATCCGTGGTGATAACTG	
ACTIN-F	CTTCATAGGAATGGAAGCTGCGGGTA	Real-Time PCR
ACTIN-R	CGACCACCTTGATCTTCATGCTGCTA	
UBIQUTIN-F	CAAGATGATCTGCCGCAAATGC	Real-Time PCR
UBIQUTIN-R	TTTAACCAGTCCATGAACCCG	
IVDH-RT-F	TATGTTCGCCAAAGAGAGCAAT	Real-Time PCR
IVDH-RT-R	GAATCACACCAGCACAATCCT	
ETFQO-RT-F	GCACTCGCCTATTCAATATCCG	Real-Time PCR
ETFQO-RT-R	GGTCCAGCATACAGAGGAAGAT	
ETFα-RT-F	ACCAGAGCTGCCGTAGATG	Real-Time PCR
ETFα-RT-R	CTCATTCCTGCCAAGTGTTGTA	
D2HGDH-RT-F	GGATGGGTAAATACAAGGGT	Real-Time PCR
D2HGDH-RT-R	AACCAGCTTCACAAGTAAGA	
REC8-RT-F	GATGATATGAAAGGGGAGTTGAGTG	Real-Time PCR
REC8-RT-R	TCACCCATTGCGGACAGAA	
MEL1-RT-F	TGACTGACAAGAGCGGAAACAT	Real-Time PCR
MEL1-RT-R	GCATCGGCTGTAAAATGGTTC	
ETFβ-Probe-F	ATGAAGATCCTAGTGGCGGT	*In situ* hybridization
ETFβ-Probe-R	ACGGTGGCGGCGACGACCTC	
MEL1-Probe-F	CCTACTATGCTCATCTTGCTGC	*In situ* hybridization
MEL1-Probe-R	CTACCAGAATATTGGGCTCTCC	
REC8-Probe-F	CTCACTCGCTCATCCATT	*In situ* hybridization
REC8-Probe-R	CATCTTTGGTCCCCTTGA	
