# Supplementary material for: Nitrogen nutrition contributes to plant fertility by affecting meiosis initiation
Source: Nat Commun. 2022 Jan 25;13:485. doi: 10.1038/s41467-022-28173-3 (PMC8789853; doi:10.1038/s41467-022-28173-3)
Supplement: Supplementary file 3 — Description of Additional Supplementary Files [file 41467_2022_28173_MOESM3_ESM.pdf]

Description of Additional Supplementary Files

File name: Supplementary Data 1

Description: The genotype of each plant in the mapping population at each of the markers tested including the first-pass mapping and fine mapping.
