# Supplementary material for: Nitrogen nutrition contributes to plant fertility by affecting meiosis initiation
Source: Nat Commun. 2022 Jan 25;13:485. doi: 10.1038/s41467-022-28173-3 (PMC8789853; doi:10.1038/s41467-022-28173-3)
Supplement: Supplementary file 5 — Reporting Summary [file 41467_2022_28173_MOESM5_ESM.pdf]

nature portfolio

Corresponding author(s): Zhukuan Cheng

Last updated by author(s): Dec 6, 2021

Reporting Summary

Nature Portfolio wishes to improve the reproducibility of the work that we publish. This form provides structure for consistency and transparency in reporting. For further information on Nature Portfolio policies, see our Editorial Policies and the Editorial Policy Checklist.

Statistics

For all statistical analyses, confirm that the following items are present in the figure legend, table legend, main text, or Methods section.

n/a	Confirmed	
☐	☒	The exact sample size (*n*) for each experimental group/condition, given as a discrete number and unit of measurement
☐	☒	A statement on whether measurements were taken from distinct samples or whether the same sample was measured repeatedly
☐	☒	The statistical test(s) used AND whether they are one- or two-sided<br>*Only common tests should be described solely by name; describe more complex techniques in the Methods section.*
☒	☐	A description of all covariates tested
☒	☐	A description of any assumptions or corrections, such as tests of normality and adjustment for multiple comparisons
☐	☒	A full description of the statistical parameters including central tendency (e.g. means) or other basic estimates (e.g. regression coefficient) AND variation (e.g. standard deviation) or associated estimates of uncertainty (e.g. confidence intervals)
☐	☒	For null hypothesis testing, the test statistic (e.g. *F*, *t*, *r*) with confidence intervals, effect sizes, degrees of freedom and *P* value noted<br>*Give P values as exact values whenever suitable.*
☒	☐	For Bayesian analysis, information on the choice of priors and Markov chain Monte Carlo settings
☐	☒	For hierarchical and complex designs, identification of the appropriate level for tests and full reporting of outcomes
☐	☒	Estimates of effect sizes (e.g. Cohen's *d*, Pearson's *r*), indicating how they were calculated

Our web collection on statistics for biologists contains articles on many of the points above.

Software and code

Policy information about availability of computer code

Data collection	cellSens Standard, Zen software were used to collect images on the microscope.
Data analysis	IBS 1.0.2, MEGA 4, SMART (http://smart.embl-heidelberg.de/index2.cgi), MAFFT (https://toolkit.tuebingen.mpg.de/mafft), ESPRIPT3 (http://espript.ibcp.fr/ESPript/ESPript/), Zen software, Adobe Acrobat DC, Microsoft Excel 2016, Graphpad prism (7.0), R (base package, 3.5.0), R (pheatmap, 1.0.12), R (MetaboAnalystR, 1.0.1), KEGG Compound databasewere (http://www.kegg.jp/kegg/compound/), KEGG Pathway database (http://www.kegg.jp/kegg/pathway.html), MSEA (metabolite sets enrichment analysis), Bio-Rad CFX Manager (V1.6.541.1028) and vario PYRO cube Software (V.1.3) were used for data analysis and statistics.

For manuscripts utilizing custom algorithms or software that are central to the research but not yet described in published literature, software must be made available to editors and reviewers. We strongly encourage code deposition in a community repository (e.g. GitHub). See the Nature Portfolio guidelines for submitting code & software for further information.

Data

Policy information about availability of data

All manuscripts must include a data availability statement. This statement should provide the following information, where applicable:

- Accession codes, unique identifiers, or web links for publicly available datasets
- A description of any restrictions on data availability
- For clinical datasets or third party data, please ensure that the statement adheres to our policy

The data supporting the findings from this study are available within the article file and its Supplementary Infromation. The additional details on map-based cloning are provided as the Supplementary Data. The source data for Fig. 3c, Fig. 5, Fig. 6, Fig. 7a, Fig. 7b and Fig. 7c are provided in the Source Data file. And the raw images of microscopy data and the metabolomic data are provided in the Source Data file. Source data are provided with this paper. The microscopy data are

available at BioStudies, and the accession number S-BSST743 [https://www.ebi.ac.uk/biostudies/submit.html]. The raw metabolomics data have been deposited in Metabolights, and the unique identifier is MTBLS3924 [www.ebi.ac.uk/metabolights/MTBLS3924]. Any remaining raw data will be available from the corresponding author upon reasonable request.

Field-specific reporting

Please select the one below that is the best fit for your research. If you are not sure, read the appropriate sections before making your selection.

☒ Life sciences ☐ Behavioural & social sciences ☐ Ecological, evolutionary & environmental sciences

For a reference copy of the document with all sections, see nature.com/documents/nr-reporting-summary-flat.pdf

Life sciences study design

All studies must disclose on these points even when the disclosure is negative.

Sample size	Fig. 1a: The mutant and WT plants were taken from paddy fields in Changping (Beijijng) after three months of planting.<br>Fig. 1c, d, e: All the anthers were blanketly taken from mutant and WT plants cultivated in Hainan and Beijing paddy fields after two months.<br>Fig. 2, Fig. 3a, Fig. 4 and Supplementary Fig. 4: All the nutritional treatment experiments were carried at least three times. All the anthers were taken from mutant and WT plants cultivated in porous ceramsite for nutritional treatment in a phytotron at a temperature of 23-35 degrees Celsius with 10 h light and 14 h dark photoperiod. And the plants were treated with three nutritional gradients for at least two weeks after 50 days of planting. Four mutant and four WT plants were planted in each pot.<br>Fig. 3b, d, e: The embryo sacs and pollens were taken from panicles at flowering period, including 36 embryo sacs from WT, 36 embryo sacs etfβ (free), 38 embryo sacs from etfβ (1N) and 36 embryo sacs from etfβ (2N).<br>Fig.7a, b: The cDNA of WT and etfβ were extracted from 4 cm (±1 cm) panicles.
Data exclusions	No data was excluded from analysis.
Replication	Each experiment was reproduced at three times (i.e. treatment with different concentrations of whole nutrients and nitrogen nutrition), and all attempts at replication were successful..
Randomization	Plants were selected randomly for nutritional treatment, sampling, imaging, measuring total N contents and metabolomic analysis.
Blinding	Phenotypic comparison of mutant anthers between Hainan and Beijing was not blinding because different typical anther sections were selected to proved that mutant phenotypes varied greatly between Hainan and Beijing.<br><br>Imaging, measuring total N contents, and metabolomic analysis were blinding and not subjective in nutritional and nitrogen treatment, because phenotypes of all mutant plants treated with the same nutritional or nitrogen gradient were consistent and stable.

Reporting for specific materials, systems and methods

We require information from authors about some types of materials, experimental systems and methods used in many studies. Here, indicate whether each material, system or method listed is relevant to your study. If you are not sure if a list item applies to your research, read the appropriate section before selecting a response.

Materials & experimental systems

n/a	Involved in the study
☒	☐ Antibodies
☒	☐ Eukaryotic cell lines
☒	☐ Palaeontology and archaeology
☒	☐ Animals and other organisms
☒	☐ Human research participants
☒	☐ Clinical data
☒	☐ Dual use research of concern

Methods

n/a	Involved in the study
☒	☐ ChIP-seq
☒	☐ Flow cytometry
☒	☐ MRI-based neuroimaging
